# A retrospective evaluation of the Euroarray STI-11 multiplex system for the detection of eight STI causing agents

**DOI:** 10.1038/s41598-023-38121-w

**Published:** 2023-07-14

**Authors:** Karl Dichtl, Andreas Osterman, Johannes Forster, Lena Jakob, Sebastian Suerbaum, Michael J. Flaig, Sören Schubert, Johannes Wagener

**Affiliations:** 1 Lehrstuhl für Medizinische Mikrobiologie und Krankenhaushygiene, Max von Pettenkofer-Institut, Medizinische Fakultät, LMU München, Munich, Germany; 2 Diagnostic and Research Institute of Hygiene, Microbiology and Environmental Medicine, Medical University of Graz, Graz, Austria; 3 Lehrstuhl für Virologie, Max von Pettenkofer-Institut, LMU München, Medizinische Fakultät, Munich, Germany; 4 Institut für Hygiene und Mikrobiologie, Julius-Maximilians-Universität Würzburg, Würzburg, Germany; 5 Klinik und Poliklinik für Dermatologie und Allergologie, Klinikum der Universität München, LMU München, Munich, Germany; 6 Department of Clinical Microbiology, School of Medicine, Trinity College Dublin, the University of Dublin, St. James’s Hospital Campus, Dublin, Ireland

**Keywords:** Diagnostic markers, Infectious diseases

## Abstract

With an incidence of more than > 1,000,000/day, sexually transmitted diseases remain a major challenge for health care systems worldwide. To reduce disease burden, complications, and spread, rapid diagnosis permitting early therapy is pivotal. The range of pathogens is wide and co-infections are common. This complicates pre-analytics, which are based on different laboratory techniques with potentially long turnaround times, e.g., cultivation and multistep serologies. Multiplex PCR provides the opportunity to overcome these limitations. In this study, we evaluated a novel assay, the Euroarray STI-11 microarray (EA; Euroimmun Medizinische Labordiagnostika), for the detection of eight obligate or facultative pathogens. Three-hundred-thirteen clinical specimens, which had been tested and pre-characterized for STI causing agents as part of routine diagnostics, were used as cases and controls in this retrospective study. The EA detected 34/44 *Chlamydia trachomatis,* 48/50 HSV-1, 50/50 HSV-2, 48/48 *Mycoplasma hominis,* 45/47 *Neisseria gonorrhoeae*, 9/11 *Treponema pallidum*, 46/46 *Ureaplasma parvum*, and 49/49 *Ureaplasma urealyticum* infections, respectively. 293 samples were EA positive, with polymicrobial infections (positive for two to six microbial or viral agents) detected in 130/293 cases. Specificities were 100% in the respective control groups (n = 18–48 depending on targeted pathogen) except for *N. gonorrhoeae* (25/26) and *U. urealyticum* (44/45). The broad spectrum of obligate and facultative pathogens targeted by the EA makes it a valuable tool in the setting of STI diagnostics and surveillance. The test has the potential to diagnose diseases neglected or overlooked in routine clinical practice. Besides a low sensitivity for *C. trachomatis*, the EA demonstrated high performance for all analyzed parameters. Further studies are warranted in order to capture a larger variety of the tested pathogens.

## Introduction

Sexually transmitted infections (STIs) remain a significant burden for healthcare systems worldwide with an estimated incidence of > 1,000,000 cases every day^1^. The (re)increase in STIs observed since the 1990s is accompanied by the new threat from resistant pathogens, e.g., extensively drug-resistant *Neisseria gonorrhoeae*^2^. The global burden of disease and the burden on the affected individual are high: a primarily localized infection can lead to serious and lifelong complications such as infertility or congenital infections resulting in permanent disability^1,2^. Therefore, timely diagnosis and targeted therapy are essential.

However, the diagnosis of STIs is complicated by various factors. For instance, only very few STI pathogens can be detected by convenient and inexpensive methods such as microscopy or culture^3–6^. Serologic assays are only available for few STIs, and often cannot provide a definite distinction between a prior and current infection, e.g., for *Chlamydia trachomatis*^7^. For the reliable direct detection of most obligate and opportunistic bacterial STI pathogens, i.e., *C. trachomatis*, *Mycoplasma genitalium*, *M. hominis*, *Treponema pallidum, Ureaplasma* spp., molecular methods (nucleic acid amplification tests, NAATs) are required^6–9^.

However, this elaborate technique needs well-equipped laboratories and skilled personnel, and there are significant costs for supply like reagents for high quality DNA preparation. This is all the more true since STIs often present as coinfection with more than one agent, with coinfection rates of 25% and higher in high-risk populations like men who have sex with men^10,11^. This makes it necessary to test for different organisms. The current development towards multiplex systems combines the molecular detection of several pathogens in one assay simultaneously, and hence addresses the issues of co-infections and cost pressure^6,8,12–16^. The number of targets detected in the multiple commercially available tests varies considerably. While some assays only focus on two pathogens, others represent a kind of diagnostic all-round approach. To date, the Euroarray STI-11 (EA; Euroimmun Medizinische Labordiagnostika, Lübeck, Germany) is the assay that covers the largest spectrum of obligate and opportunistic STI pathogens including a total of 11 bacterial, viral, and protozoan agents: *C. trachomatis*, *Haemophilus ducreyi*, HSV-1, HSV-2, *M. genitalium*, *M. hominis*, *N. gonorrhoeae*, *T. pallidum, Trichomonas vaginalis*, *Ureaplasma parvum*, and *Ureaplasma urealyticum*. Following multiplex amplification, the reaction mix is processed via the previously described Euroarray system (Euroimmun) including hybridization to a multiarray chip and fluorescence analysis^17^. The EA was CE certified and launched to the market in 2017.

However, to date, only one very recent study has evaluated the performance of the EA test system focusing on only few pathogens and with a limited number of samples, e.g., only three cases of *Chlamydia* infection and one case of gonorrhea^18^. Therefore, this retrospective study aimed to assess sensitivities and specificities for the detection of eight STI causing agents (*C. trachomatis,* HSV-1, HSV-2, *M. hominis, N. gonorrhoeae, T. pallidum*, *U. parvum,* and *U. urealyticum*).

## Materials and methods

### Specimens

The 313 clinical specimens included in this retrospective study were contributed by four diagnostic laboratories, two of which are located at the Max von Pettenkofer Institute (Department of Virology and Department of Medical Microbiology and Hospital Epidemiology), Munich, Germany. In addition, the Institute for Medical Microbiology and Hygiene of the University of Würzburg (Würzburg, Germany) and the diagnostic laboratory of the Department of Dermatology and Allergy, University Hospital, LMU Munich (Munich, Germany) participated in the study. The samples dated from the years 2011 to 2019. Of the 313 specimens, 131 (41.9%) were obtained from female patients and 103 (32.9%) from male patients. No information about sex was available for the remaining 73 cases (23.3%).

For none of the specimens, reference results for all 11 targets of the EA were available. Typically, only the test result for one target (representing the inclusion criterion for this study) was known to the investigators.

The vast majority of specimens (85.3%) had been pre-characterized by different commercially available PCRs and in-house PCRs in the respective study centers. The CE certified assays were the GeneProof *Chlamydia trachomatis* and the GeneProof *Neisseria gonorrhoeae* PCR kits (GeneProof, Brno, Czech Republic), which were performed on a Rotorgene Q cycler (Qiagen, Venlo, Netherlands) for the diagnosis of the respective pathogens. For the detection of HSV-1 and HSV-2, previously described in-house PCRs performed on ABI 7500 Fast Real-Time cyclers were used (Applied Biosystems, Foster City, USA)^19,20^. The characteristics of the in-house PCRs for the diagnosis of *Ureaplasma* spp., *M. hominis* and *C. trachomatis* and of the *N. gonorrhoeae*, which were performed using the BDmax system (Becton, Dickinson and Company, Franklin Lakes, USA), are specified in Supplementary Table S1. A minority of 18% of specimens were pre-characterized by cultivation to be positive for *N. gonorrhoeae* or *M. hominis* (Supplementary Table S2). The syphilis cohort included thirteen biopsies with pathognomonic signs in histopathology.

The analyzed specimens were mostly swabs (n = 215), which were typically obtained using the eSwab system (nylon flocking, in liquid AMIES medium; Copan, Brescia, Italy) or, to a lesser extent, using the Mastaswab system (rayon flocking, solid AMIEs medium; Mast, Bootle, UK). Biopsies (n = 14) and fluids (n = 6) such as urine or ejaculate made up only a small percentage. No information concerning the nature of the specimen was available for the remaining samples (n = 78). The most common sampling site was the urogenital tract (n = 136), followed by the skin, the oropharyngeal and the anorectal region (n = 46, 21, and 12). For 98 specimens, information about the sampling site was not accessible for this study.

### Sample processing

Depending on the respective study site, different protocols for DNA isolation were applied, including manual DNA preparation. However, by far most of the samples were processed fully automated using either the QIAsymphony system (Qiagen) or the MagNA Pure system (Roche, Rotkreuz, Switzerland).

The EA was performed according to the manufacturer’s instructions (Euroimmun Medizinische Labordiagnostika, Lübeck, Germany). Briefly, sample processing involved three steps: (1) multiplex PCR in a Mastercycler nexus SX1 block cycler (Eppendorf, Hamburg, Germany), (2) hybridization of the amplicons to a microarray chip (60′ 45 °C), and (3) read-out for pathogen-specific DNA and a set of controls (DNA, amplification, hybridization, cross contamination). Fluorescence intensity of the microarray was measured with the EUROArrayScanner system, and the results were evaluated by the EUROArrayScanner software (Euroimmun Medizinische Labordiagnostika) according to the specifications of the manufacturer.

Two conditions can cause invalid results in the EA test system: first, when the DNA control (target: human HSP90AB1 gene) is negative, pathogen-specific PCRs without amplification/hybridization cannot be assessed negative but invalid. However, detection of pathogen-specific DNA may still be reported. Second, for each individual PCR there are two hybridization sites on the microarray chip, which both need to show concordant fluorescence patterns for a valid result. Otherwise, the specific test for the respective target must be considered invalid without consequences for the other PCRs. Concordant positive and negative results upon valid controls are considered positive and negative, respectively.

### Resolution of discordant results

In cases of discrepancies between PCR-based reference results and EA results, a third PCR was performed and its result was considered accurate. For detection of *C. trachomatis, M. hominis, N. gonorrhoeae, U. parvum* and *U. urealyticum*, the CE certified Seegene test system using the Allplex STI Essential assay (Seegene, Seoul, South Korea) was used. Discordant HSV and *T. pallidum* results were analyzed using previously described in-house PCRs (cyclers: ABI 7500 Fast Real-Time PCR system for HSV and Tgradient cycler [Analytik Jena, Jena, Germany] for *T. pallidum* PCRs)^19–21^.

In one case, there was not enough specimen/DNA left to perform a decisive third PCR resulting in exclusion of the sample from the study.

### Ethics statement

This retrospective study adheres to the principles of the Declaration of Helsinki and was approved by the ethics committees of the University Hospital of LMU Munich (Ethikkommission der Medizinischen Fakultät der LMU München) and of the University Hospital of Würzburg, and a waiver of informed consent was granted by these ethics committees. For this study, sample processing of the pre-characterized specimens and data analysis were performed anonymously.

## Results

### Rate of invalid tests

For this study, 313 pre-characterized samples were analyzed using the EA test system. The assay did not yield any invalid results due to discordant results between the two hybridizations of one specific target. However, in 25 samples the DNA control was negative (validity rate of 92%), which impeded the assessment of the respective non-positive pathogen specific PCRs. Nevertheless, 24 of the 25 samples still yielded positive results for one or more pathogens since amplification of specific DNA was detectable. This suggests that the invalid results are primarily due to pre-analytical limitations, i.e., inadequate sampling, resulting in nearly cell-free specimens.

### Positivity rate and incidental findings

Twenty-one EA tests did not detect any pathogen-specific DNA with one test being invalid due to a negative DNA control. Thirteen of the samples negative for all 11 STI-targets were part of the *T. pallidum* control group, with the remaining nine *T. pallidum* control specimens being positive for at least one PCR. Seven specimens were false negative according to the results of the reference method. In total, positive results were obtained in 94% (n = 293) of the included samples. In nearly half of the positive samples (130 of 293), more than one pathogen was detected. The most common microbes were *U. parvum* (n = 84), *U. urealyticum* (n = 81), and *N. gonorrhoeae* (n = 63) (Supplementary Table S3).

Since the samples were only selectively pre-characterized for the 11 targets of the EA, we encountered a significant number of incidental findings, i.e., detection of pathogens, which were not known to be present in the respective sample prior to EA analysis. *Ureaplasma* spp. and *Mycoplasma* spp. (74 and 41 of 151 incidental findings) were the most prevalent incidental findings. While these bacteria are not necessarily the cause of symptoms or disease, other microbes are considered obligate pathogens, e.g., *N. gonorrhoeae* and *T. pallidum,* which were incidental EA findings in 17 and two samples, respectively (Supplementary Table S3). This study did not include any samples that were pre-characterized to contain DNA of *H. ducreyi, M. genitalium,* or *T. vaginalis*. However, incidental findings occurred for *M. genitalium* (nine positive samples) and *T. vaginalis* (three positive samples).

### Discrepant results

In 17% of specimens (53 of 313), discordant results between the EA and the respective reference method (PCR in 52 cases and culture in 1 case) were obtained. EA positivity was unexpected in 19 and EA negativity in 33 cases. A third independent and decisive PCR using the same DNA preparation was performed for the 52 cases with discrepant PCR results. This re-test verified the EA result in 34 and the reference result in the remaining 18 cases (65% and 35% of discrepant results, respectively). Upon discordant results, EA positivity was confirmed in 89% of cases (17/19) and EA negativity in 55% of cases (18/33).

### Performance of the specific PCRs

#### C. trachomatis

Two laboratories contributed a total of 88 samples tested for *C. trachomatis* by different PCRs (in-house and CE certified assays). Eighty-five specimens yielded valid results in the EA. Surprisingly, 16 (33%) of the 44 samples, which were pre-characterized to be positive, were EA negative. These specimens were re-tested with a CE certified multiplex PCR assay. The reference result was confirmed in 10 and the EA result in six cases. One sample pre-characterized to be negative was tested positive in the EA and the subsequently performed decisive PCR. In summary, while the specificity for *C. trachomatis* was 100%, the sensitivity was only 77% (Table 1).

**Table 1:** Sensitivities and specificities of the Euroarray test system.

Target	True pos	(%)	True neg	(%)
*C. trachomatis*	34/44	(77)	41/41	(100)
HSV-1	48/50	(96)	45/45	(100)
HSV-2	50/50	(100)	44/44	(100)
*M. hominis*	48/48	(100)	48/48	(100)
*N. gonorrhoeae*	45/47	(96)	25/26	(96)
*T. pallidum*	9/11	(82)	18/18	(100)
*U. parvum*	46/46	(100)	44/44	(100)
*U. urealyticum*	49/49	(100)	44/45	(98)

True positivity (pos.) and true negativity (neg.) rates of the EA refer to the results of the respective reference methods applied in this study or (in cases of discrepancies between the EA and the reference method) to the results of an independent decisive third test.

#### Herpes simplex (HSV-1, HSV-2)

Fifty positive samples for each pathogen served as case group for the respective virus. In parallel, the HSV-1 case group was exploited as control group for the HSV-2 test and vice versa. Since some samples yielded negative results in the DNA control, all negative pathogen PCRs had to be excluded (invalid results). Hence, 45 and 44 samples remained in the HSV-1 and HSV-2 negative control group, respectively. The EA had a 100% specificity for both Herpes viridae. While all HSV-2 cases were detected by the EA, two HSV-1 positive samples yielded a false negative result (sensitivity of 96%). Both samples were characterized by very low viral loads: the first one contained only 130 genome equivalents (geq) according to the applied quantitative PCR (qPCR), and the amount of DNA in the second sample was even under the qPCR’s limit of quantification (< 10 geq/reaction).

#### M. hominis

Forty-eight *M. hominis-*positive and 48 M*. hominis-*negative samples from two study sites were tested with the EA without any discrepancies (sensitivity and specificity of 100% each) (Table 1).

#### N. gonorrhoeae

Forty-seven samples characterized as *N. gonorrhoeae*-positive sampled at three centers were included. The EA yielded positive results in 45 of 47 cases (sensitivity of 96%). Of the two samples with discordant results, one originally yielded growth of *N. gonorrhoeae*, the other was identified via PCR. The latter was re-tested with a CE certified multiplex PCR assay, i.e., the Allplex STI Essential kit, which confirmed the reference result. In both EA-negative samples, hybridization of specific amplicons was detectable, but the fluorescence remained below the cut-off. A concordant negative test result was obtained in 25 of 26 samples (reference method: in-house PCR and/or culture; specificity of 96%). The Allplex STI Essential kit again proofed the reference result in the discrepant case.

#### T. pallidum

One study site contributed DNA isolated from 13 paraffin embedded tissues, which displayed histopathologic morphology suspicious for syphilis. Seven samples were positive for *T. pallidum* in the EA. The remaining six DNAs were analyzed with another *T. pallidum* specific PCR that was positive in only two of the samples. Two specimens that were not pre-characterized yielded a positive result in the EA and in the subsequently performed specific PCR. Hence, the EA was able to establish the diagnosis of syphilis in 9/11 cases (82%). As control group, we identified 22 patients that had no serologic evidence for a *T. pallidum* (re-)infection in sera obtained 3–15 weeks after sampling of urogenital or anorectal specimens. Those were all tested negative in the EA (specificity of 100%).

#### *Ureaplasma* spp.

One laboratory contributed 90 and 94 samples, which had been analyzed for *U. parvum* and *U. urealyticum*, using in-house PCRs. The EA yielded concordant positive results for all 47 and 49 positive *U. parvum* and *U. urealyticum* samples, respectively (sensitivities of 100% each). All 44 *U. parvum* negative samples were tested true negative by the EA (100% specificity). One of the 45 samples pre-characterized as *U. urealyticum* negative was tested positive by the EA, which was refuted by a third PCR, resulting in a specificity of 98%.

## Discussion

Microbiological multiplex tests can show their strength particularly in two situations: firstly, when the probability for polymicrobial infections is high, and secondly, when several different pathogens must be ruled out as a cause of the symptoms. It is precisely this scenario that we encounter within the field of STI diagnostics. One STI rarely comes alone, and therefore, patients at risk typically benefit from a comprehensive diagnostic approach: for instance, both IDSA and EACS guidelines recommend testing for syphilis, gonorrhea, and *C. trachomatis* infection upon primary HIV infection^22,23^. Multiplex assays enable laboratories to perform several analyses in one procedure resulting in resource-saving and rapid results. The diagnostic all-around approach supports physicians, who are less experienced in recognizing and managing STIs, thereby ensuring the quality of patient care. However, even skilled infectious diseases specialists happen to misidentify an atypically presenting common disease or a rare and neglected disease.

On the downside, multiplex assays have certain limitations, which need to be considered. First, one should ask whether NAAT represents an adequate tool to diagnose the disease. To stay with the above example of STI screening in the setting of primary HIV infection: in contrast to gonorrhea and *C. trachomatis* infection, syphilis is primarily diagnosed via serologic methods, which do not rely on the presence of accessible sampling sites containing a reasonable pathogen load, e.g., chancres^24,25^. A prerequisite here is that the corresponding pathogens are present at the same sampling site of the smear. In the case of syphilis and herpes infections in particular, a negative smear can lead inexperienced physicians to be lulled into a false sense of security. Notably, there is a trend in recently published guidelines to strengthen the role of NAAT for the early diagnosis of syphilis: for instance, different European medical associations now additionally recommend the molecular detection of *T. pallidum* from ulcerations^25–27^.

The great strength of a multiplex system, i.e., the ability to detect many targets in one assay, can also be weakness. The test generates a multitude of results, whose medical relevance is questionable. In many cases, attending physicians are only interested in a subset of results but could eventually be presented with positive test results that were not intentionally requested. While the detection of obligate pathogens such as *N. gonorrhoeae* or *T. pallidum* always indicates diseases that require treatment, other organisms could in many cases represent harmless commensals. Incidental findings of a multiplex-based diagnostic approach can therefore lead to unnecessary treatment with the risk of adverse events. In fact, the most frequent incidental findings in the present study, i.e., detection of DNA of germs that were not known to the study investigators in advance, were *M. hominis* and *Ureaplasma* spp. Currently, the question of the need for treatment of these germs is still a matter of debate and was not addressed by international evidence-based management guidelines^9,28^. Well-designed, large-scale studies will be necessary to elucidate the benefit of diagnosing *M. hominis* and *Ureaplasma* spp. Therefore, one could argue that upon using the EA only obligate pathogens should be reported for clinical use, whereas information about the detection of *M. hominis* and *Ureaplasma* spp. should serve surveillance purposes only. Considering the ongoing developments in NAAT-based diagnostics, which will lead to more and more positive findings, better epidemiological data is needed to assess the medical relevance for the individual patient. Multiplex methods such as the one studied here could be a helpful tool for surveillance, but is also a challenge for the novel field of diagnostic stewardship^29^.

Twenty-four of 25 invalid EA assays were characterized by a negative DNA control but positive pathogen-specific PCRs. This finding suggests insufficient sampling resulting in specimens scarce of cells. It is a major disadvantage of culture, that a negative result does not provide information concerning the quality of the sample. Contrarily, negative DNA controls of NAAT allow the differentiation between negative and invalid results.

For the majority of evaluated parameters, EA sensitivity and specificity were 100%. All samples yielding DNA of HSV-2, *M. hominis, U. parvum,* and *U. urealyticum*, respectively, were identified. Yassin et al*.* reported in their recent evaluation comparable results for these three agents except for notably lower specificities for *M. hominis* (84%) and *U. parvum* (88%)^18^. However, the limitations of this result have to be considered, i.e., that 1) the second evaluated commercial assay (Allplex STI essential) also displayed low specificities (79% and 94%) and that 2) the true nature of the result was solely defined by the single result of an *in house* PCR. Performance for the detection of other pathogens was either not investigated or relied on less than 10 samples, which restricts further comparison with our study. The missed detection of few cases of *N. gonorrhoeae* and HSV-1 in our study can likely be attributed to low DNA load (sensitivities of 96% each). For instance, according to the manufacturer, the lower limit of detection of HSV-1 DNA is 50 geq/reaction, and one of the EA false negative HSV-1 samples yielded less than 10 geq/reaction of our reference standard (in-house qPCR). Also, *N. gonorrhoeae* DNA was still amplified in the false negative samples of gonorrhea patients, but the cut-off has just not been exceeded.

In contrast to those good to excellent results, the sensitivity for detection of *C. trachomatis* was surprisingly low (77%). The only other study investigating the performance of the EA did not report a similar result, but only included three samples that were pre-characterized as *C. trachomatis* positive^18^. However, this weakness in diagnosing that specific pathogen has also been reported for other multiplex assays before: De Salazar and colleagues found a sensitivity of only 84% for the Allplex STI Essential, which was used to resolve discrepancies in this study^13^. The reference method used for comparison in the work of De Salazar et al*.* was the Aptima Combo 2 test (Hologic, San Diego, CA, USA), a transcription-mediated amplification (TMA) assay for the synchronous detection of *C. trachomatis* and *N. gonorrhoeae*. Interestingly, again significant rates of false negative *C. trachomatis* results were reported also for this assay in two Northern European studies^30,31^. However, this finding can be attributed to the prevalence of the Finnish new variant of *C. trachomatis* characterized by the C1515T mutation in the 23S rRNA gene, which is the target of the Aptima Combo 2 assay. Contrarily, the EA targets the ompA gene (NCBI accession number EU296817) coding for the major outer membrane protein MOMP, which is also the basis of serotyping^32^. One could speculate that the manufacturer’s primer design might disfavor the detection of specific ompA sequences, which were frequently included in our study. Considerably, other commercially available PCRs target more than one genomic region, e.g., a sequence located on a cryptic plasmid combined with ompA or the 16S rRNA gene^33,34^. This might explain a higher sensitivity of other assays despite the low EA limit of only 20 geq/reaction specified by the manufacturer. Furthermore, the significance of the selection of the samples should not be underestimated: *C. trachomatis* load depends on demographic factors, e.g., sex and age, on medical conditions like HIV-coinfection or repeated *C. trachomatis* infections, and the anatomical site^35,36^. For instance, the pathogen load can be assumed 100-fold higher in vaginal than in oropharyngeal swabs^35^. Unfortunately, for the laboratory providing the majority of *C. trachomatis* positive samples, information concerning sampling site and demographic characteristics was not available due to medical data protection. Therefore, we cannot exclude the possibility that the nature of the selected samples may adversely affect the performance of the EA. Other explanations for the false-negative results include instability of the target or competition issues in the multiplex reaction.

This should also be considered in the analysis of the test performance in the setting of syphilis. According to the manufacturer, the *T. pallidum* specific PCR was validated in swabs. In this study, the majority of *T. pallidum* positive samples and both EA false negative samples were biopsies. However, due to the low number of cases of syphilis included in this study (n = 11), our results must be interpreted with caution.

This study has some limitations that have to be considered: 1) an overall low case number, 2) the lack of clinical information, i.e., presence of symptoms, and 3) the selection of samples. The latter factor may cause selection bias distorting the evaluation of test performance. Considerably, the EA is disadvantaged by the study design per se, which makes the assay compete against other tests that were defined to be the respective reference method.

In summary, we were able to demonstrate the feasibility of this test to detect most of the included targets: besides a low sensitivity for *C. trachomatis*, the EA demonstrated high performance. Further studies are warranted in order to capture a larger variety of the tested pathogens. The EA might be a valuable tool for the diagnosis or surveillance of STI thanks to the broad spectrum of detected obligate and facultative pathogens.

### Key findings

The Euroarray STI-11 multiplex assay demonstrated high sensitivities and specificities for the detection of HSV-1, HSV-2, *Mycoplasma hominis, Neisseria gonorrhoeae, Treponema pallidum, Ureaplasma parvum*, and *Ureaplasma urealyticum*.

The sensitivity for the detection of *Chlamydia trachomatis* infection was only 77%.

The assay is a helpful tool for surveillance and to detect polymicrobial infections.

## Supplementary Information

Supplementary Table 1.

Supplementary Table 2.

Supplementary Table 3.

## Data Availability

The datasets generated and analyzed during the current study are available from the corresponding author on reasonable request.
