# Supplementary material for: A retrospective evaluation of the Euroarray STI-11 multiplex system for the detection of eight STI causing agents
Source: Sci Rep. 2023 Jul 14;13:11382. doi: 10.1038/s41598-023-38121-w (PMC10349140; doi:10.1038/s41598-023-38121-w)
Supplement: Supplementary file 1 — Supplementary Table 1. [file 41598_2023_38121_MOESM1_ESM.docx]

**Supplementary table 1. Characterization of in-house PCRs.** Specification of target locus (with accession number), analytical sensitivity (in copies / ml), specificity (%), and number of study samples analyzed using the respective assay (n and % of all samples pre-characterized positive for the pathogen). sens., analytical sensitivity; spec., specificity; cp. / ml, copies per milliliter;

| target organism | target locus  (accession No.) | cycler | sens.  (cp./ml) | spec.  (%) | pos. samples pre-characterized by the assay | |
| --- | --- | --- | --- | --- | --- | --- |
| *C. trachomatis* | cryptic plasmid  (CP010572) | BDmax | 10^2^ | 100 | 4 | (9 %) |
| *M. hominis* | *gap* (GAPDH)  (AJ243692) | BDmax | 10^2^ | 100 | 46 | (84 %) |
| *N. gonorrhoeae* | cryptic plasmid  (CP003910) | ABI 7500  Fast | 10^1^ | 100 | 12 | (26 %) |
| *U. parvum* | urease complex  (AF085731) | BDmax | 10^2^ | 100 | 46 | (100 %) |
| *U. urealyticum* | urease complex  (AF085720) | BDmax | 10^2^ | 100 | 49 | (100 %) |
