# Supplementary material for: A retrospective evaluation of the Euroarray STI-11 multiplex system for the detection of eight STI causing agents
Source: Sci Rep. 2023 Jul 14;13:11382. doi: 10.1038/s41598-023-38121-w (PMC10349140; doi:10.1038/s41598-023-38121-w)
Supplement: Supplementary file 2 — Supplementary Table 2. [file 41598_2023_38121_MOESM2_ESM.docx]

**Supplementary table 2. Characterization of cultivation and identification techniques applied for pre-characterization of specimens.** *N. gonorrhoeae* was cultivated on Thayer-Martin agar (PolyViteX VCAT3, bioMérieux, Marcy-L’Étoile, France) in a humidified atmosphere containing 5 % CO_2_ and identified via MALDI-TOF analysis (n = 13; Biotyper, Bruker, Billerica, USA) or via biochemical differentiation (n = 12; API NH, bioMérieux). *M. hominis* was detected via phenotypical analysis after cultivation on modified PPLO agar (20 % horse serum, 4 % blood agar base, 1 % urea, 0.6 % phenol red, 0.3 % glucose, 0.1 g ampicillin, 0.03 % manganese sulfate, adjusted to a pH of 6.7).

| organism | culture medium | identification technique | no. of samples | |
| --- | --- | --- | --- | --- |
| *N. gonorrhoeae* | Thayer-Martin agar | MALDI-TOF | 13 | 4 % |
| *N. gonorrhoeae* | Thayer-Martin agar | API NH | 12 | 4 % |
| *M. hominis* | modified PPLO agar | phenotypical analysis | 8 | 3 % |
