# Supplementary material for: A retrospective evaluation of the Euroarray STI-11 multiplex system for the detection of eight STI causing agents
Source: Sci Rep. 2023 Jul 14;13:11382. doi: 10.1038/s41598-023-38121-w (PMC10349140; doi:10.1038/s41598-023-38121-w)
Supplement: Supplementary file 3 — Supplementary Table 3. [file 41598_2023_38121_MOESM3_ESM.docx]

**Supplementary table 3. Results of the Euroarray test system.** The percentages refer to the total number of samples included (n = 313). Incidental finding (inc. find.) is defined as detection of a microbe / virus in a sample, which was either not previously characterized to test positive for the respective agent or for which information about the presence of the respective agent was not known to the study laboratory. Inf., infection.

|  | | n | % | inc. find. (n) |
| --- | --- | --- | --- | --- |
| all tests | | 313 | 100 | 102 |
| positive tests | | 293 | 94 | 96 |
|  | *C. trachomatis* | 39 | 12 | 6 |
|  | *H. ducreyi* | 0 | 0 | 0 |
|  | HSV-1 | 52 | 17 | 4 |
|  | HSV-2 | 54 | 17 | 4 |
|  | *M. genitalium* | 9 | 3 | 9 |
|  | *M. hominis* | 79 | 25 | 32 |
|  | *N. gonorrhoeae* | 63 | 20 | 17 |
|  | *T. pallidum* | 9 | 3 | 2 |
|  | *T. vaginalis* | 3 | 1 | 3 |
|  | *U. parvum* | 84 | 27 | 38 |
|  | *U. urealyticum* | 81 | 26 | 36 |
| monomicrobial inf. | | 162 | 52 | - |
| polymicrobial inf. | | 130 | 42 | - |
|  | 2 microbes / viruses | 94 | 30 | - |
|  | 3 microbes / viruses | 27 | 9 | - |
|  | 4 microbes / viruses | 4 | 1 | - |
|  | 5 microbes / viruses | 4 | 1 | - |
|  | 6 microbes / viruses | 1 | < 1 | - |
